# Uropathogenic *Escherichia coli* pathogenicity islands and other ExPEC virulence genes may contribute to the genome variability of enteroinvasive *E. coli*

**DOI:** 10.1186/s12866-017-0979-5

**Published:** 2017-03-16

**Authors:** Laís Cristina da Silva, Ana Carolina de Mello Santos, Rosa Maria Silva

**Affiliations:** Department of Microbiology, Immunology, and Parasitology, Federal University of São Paulo, Rua Botucatu 862, CEP 04023-062 São Paulo, SP Brazil

**Keywords:** Enteroinvasive *E. coli*, Uropathogenic *E. coli*, Phylogenetic origin, Pathogenicity-associated islands, ExPEC virulence genes

## Abstract

**Background:**

Enteroinvasive *Escherichia coli* (EIEC) may be the causative agent of part of those million cases of diarrhea illness reported worldwide every year and attributable to *Shigella*. That is because both enteropathogens have many common characteristics that difficult their identification either by traditional microbiological methods or by molecular tools used in the clinical laboratory settings. While *Shigella* has been extensively studied, EIEC remains barely characterized at the molecular level. Recent EIEC important outbreaks, apparently generating more life-threatening cases, have prompted us to screen EIEC for virulence traits usually related to extraintestinal pathogenic *E. coli* (ExPEC). That could explain the appearance of EIEC strains presenting higher virulence potential.

**Results:**

EIEC strains were distributed mainly in three phylogroups in a serogroup-dependent manner. Serogroups O124, O136, O144, and O152 were exclusively classified in phylogroup A; O143 in group E; and O28ac and O29 in group B1. Only two serogroups showed diverse phylogenetic origin as follows: O164 was assigned to groups A, B1, C, and B2 (one strain each), and O167 in groups E (five strains), and A (one strain) (Table 1). Eleven of 20 virulence genes (VGs) searched were detected, and the majority of the 19 different VGs combinations found were serogroup-specific. Uropathogenic *E. coli* (UPEC) PAI genetic markers were detected in all EIEC strains. PAIs I_J96_ and II_CFT073_ were the most frequent (92.1 and 80.4%, respectively). PAI IV_536_ was restricted to some serogroups from phylogroups A, B1 and E. PAI I_CFT073_ was uniquely detected in phylogroups B2 and E. A total of 45 (88%) strains presented multiple PAI markers (two to four). PAIs I_J96_ and II_CFT073_ were found together in 80% of strains.

**Conclusions:**

EIEC is a DEC pathovar that presents VGs and pathogenicity island genetic markers typically associated with ExPEC, especially UPEC. These features are distributed in a phylogenetic and serogroup-dependent manner suggesting the existence of stable EIEC subclones. The presence of phylogroups B2 and E strains allied to the presence of UPEC virulence-associated genes may underscore the ongoing evolution of EIEC towards a hypervirulent pathotype.

## Background

Enteroinvasive *Escherichia coli* (EIEC) is a diarrheagenic *E. coli* (DEC) pathotype that shares its pathogenic behavior with *Shigella* so that they have been considered a unique pathovar [1]. Like the other DEC pathotypes EIEC infections occur by the fecal-oral route, and usually are more prominent in low-income regions due to poor sanitation [2–4]. Nevertheless, outbreaks associated with contaminated food and water can occur among high-income people groups as well [5–9].

The pathogenicity of EIEC/*Shigella* is dependent on genetic virulence determinants coded by genes frequently located in *p*athogenicity-*a*ssociated *i*slands (PAIs) usually dispersed both on the chromosome and in the invasion plasmid (pINV), present in all virulent EIEC strains [10–13].

Compared to other DEC, EIEC is referred as a rare etiological agent of endemic diarrhea while *Shigella* is estimated to cause millions of cases annually worldwide, with a mortality rate that can reach 1% among children groups [14]. This discrepancy may be due to the absence of simple laboratory methods to differentiate EIEC from *Shigella* since both shares almost all the characteristics used in their identification. With all these common features, it is plausible that misidentification occurs, and many of those million *Shigella* cases may be in fact caused by EIEC.

The use of modern approaches like multilocus enzyme electrophoresis (MLEE), multilocus sequence typing (MLST), and comparative genomics [12, 15–17] is bringing new comprehension to the phylogenetic origin and evolution of the pathogenic and commensal lifestyles of the microorganisms in general. Clermont et al. [18] have developed a simple multiplex PCR method which has allowed assessing the phylogenetic origin of great numbers of *E. coli* isolates in epidemiological surveys all over the world. A further improved method based on the genotype of genes *arpA*, *chuA*, *yjaA* and TspE4.C2 was able to correctly assign more than 95% of *E. coli* strains into eight phylogroups (A, B1, B2, C, D, E, F, and clade I) [19].

Studies using these various approaches have confirmed that *E. coli* have a clonal structure [20] and that non-pathogenic strains differ from diarrheagenic *E. coli* (DEC), and extraintestinal pathogenic *E. coli* (ExPEC) in respect to their phylogenetic origin. There is common sense that both DEC and ExPEC have evolved in expenses of blocks of genes acquisition by strains with a permissive phylogenetic background. Those gene blocks, generally originated from non-related microorganisms, were moved to *E. coli* via horizontal gene transfer mechanisms mediated by mobile genetic elements such as PAIs, plasmids, and bacteriophages. After all, the set of foreign genes has established in specific genetic backgrounds given rise to different *E. coli* pathovars with EIEC being one of them.

While there is an extensive literature covering the details of *Shigella* genetic virulence determinants, these aspects have been poorly exploited in EIEC. The recent occurrence of two important outbreaks in Europe due to an EIEC strain [9, 21] has motivated studies on the molecular characterization and comparative genomics of EIEC strains isolated in various geographic regions to better understand the evolution of this *E. coli* pathovar [12, 17]. However, a possible way of EIEC rearrangements driven by the acquisition of mobile genetic elements engaged in virulence of other *E. coli* pathovars has not been addressed yet. That could explain the appearance of EIEC strains presenting higher virulence potential.

In this work, we examined a comprehensive set of EIEC strains encompassing most of the frequently isolated serogroups to assess their phylogenetic origin as well as carriage of virulence genes (VGs) and PAIs genetic markers considered to be involved in ExPEC pathogenicity. We have found that EIEC strains are distributed in various phylogroups in a serogroup-dependent manner. Besides, they may harbor several VGs and genetic PAI markers usually associated with uropathogenic *E. coli* (UPEC). The simultaneous carriage of extra-intestinal and diarrheagenic virulence traits by EIEC could play a role in their evolution towards hypervirulent clones. Such a possibility may turn out to be a matter of public health concern and deserves further investigation.

## Results

### Serogroup confirmation

The studied strains encompassed nine of the most frequently isolated EIEC serogroups as follows: O28ac (three strains), O164 (four strains), O29 and O144 (five strains each), O124, O136, O152, and O167 (six strains each), and O143 (10 strains) (Table 1).

**Table 1:** Main biochemical characteristics of EIEC according to serogroup and phylogroup

Serogroup	No. of strains	Phylogroup	Lactose fermentation	Motility	Lysine decarboxylation	Indole production
O28ac	1	B1	+	-	-	-
	2	B1	+	-	-	+
O29	5	B1	+	-	-	+
O124	4	A	-	-	-	-
	2	A	-	+	-	+
O136	6	A	-	-	-	-
O143	10	E	+	-	-	+
O144	5	A	+	-	-	+
O152	6	A	-	-	-	+
O164	1	B2	+	-	-	+
	1	C	+	-	-	+
	1	B1	-	-	-	+
	1	A	-	-	-	+
O167	5	E	+	-	-	-
	1	A	+	-	-	+^w^

^w^weak reaction

### EIEC main biochemical characteristics

As is usual for EIEC, all strains did not decarboxylate lysine and were non-motile, exception done to two O124 strains exhibiting flagellar type H30. Typical *E. coli* biochemical behavior in respect to lactose fermentation and indole production was serogroup-related, and uniformly observed for O29, O143, and O144, but not for O124, O136, and O152 which did not ferment lactose. All O136, one O28ac, and most O124 and O167 strains did not produce indole. Serogroup O164 was the only variable regarding lactose fermentation (two positives and two negative strains) (Table 1).

### Presence of invasion plasmids

All strains presented a variable number of plasmid bands, but all of them possessed a high molecular weight (MW) DNA-band ranging from 180 to 220 Kb compatible with the MW described for invasion plasmids (pINV) [10, 13].

### Invasive phenotype

All strains were able to invade HeLa cells in the gentamicin protection assay, confirming the invasive phenotype and showing the stability of the genes necessary for cell culture invasion even after long periods of storage.

### Determination of phylogenetic origin

By using the quadruplex PCR method [19], EIEC strains were distributed within five of the eight phylogenetic groups, with all strains in a given group presenting a uniform quadruplex (*arpA*, *chuA, yjaA,* and TspE4.C2) genotype. The frequency of phylogroups and their quadruplex genotypes were: 49% from phylogroup A (+ ˗ ˗ ˗), 29% from phylogroup E (+ + ˗ ˗), 18% from phylogroup B1 (+ ˗ ˗ +). Groups C (+ ˗ + ˗) and B2 (˗ + + +) were represented by one strain each. It is worth mentioning that all but two serogroups were strictly distributed in a given phylogroup as follows: strains from serogroups O124, O136, O144, and O152 were from phylogroup A, while O28ac and O29 were from phylogroup B1, and O143 from phylogroup E. Serogroup O167 strains were most originated from phylogroup E (83%) with only one strain typed as group A. A broad divergence was found only in serogroup O164 that was dispersed in phylogroups A, B1, B2, and C (one strain each) (Table 1).

### Presence of ExPEC virulence markers

As many as 11 out of 20 searched ExPEC VGs were found in EIEC strains in this survey. The most frequent were *ompA* (100%), *traT* (86.3%), and *sitA* (80.4%). Four VGs were present less frequently, namely: *iha* (54.9%), *irp2* (37.2%), *chuA* (31.3%), and *fimA* (27.4%). The remaining four VGs were rarely found: *kpsMTII* (3.9%), *hra*, *vat*, and *ompT* (1.9% each) (Table 2). None EIEC strain presented VGs *papA/C, sfaDE, afaBCIII, cvaC, iucD, iroN, sat*, and α-*hlyA*. Table 2 shows the genetic virulence profile found among the EIEC serogroups according to their phylogenetic origin. Serogroups O29 and O144 were uniform in their virulence profile. Nevertheless, all other serogroups, except for O164, presented a predominant set of VGs of their own, encompassing 67% to 80% of the strains in each serogroup. The absence of *sitA* and *iha* was responsible for the diversity observed in three of those serogroups (O24, O136, and O143) (Table 2).

**Table 2:** Genetic virulence profile of EIEC according to phylogroup and serogroup

Phylogroup {No. (%) of strains}	Serogroup (Total No. of strains)	Genetic virulence profile^a^	No. (%) of strains
A {25 (49)}	O124 (6)	*ompA, traT, irp2, sitA, iha*	4 (66.5)
		*ompA, traT, irp2, sitA*	1 (16.5)
		*ompA, traT, irp2*	1 (16.5)
	O136 (6)	*ompA, traT, sitA, iha*	4 (67.0)
		*ompA, traT, iha*	1 (16.5)
		*ompA, traT, sitA*	1 (16.5)
	O144 (5)	*ompA, traT, sitA, fimA*	5 (100)
	O152 (6)	*ompA, traT, irp2, sitA*	4 (67.0)
		*ompA, irp2, sitA*	1 (16.5)
		*ompA, traT, irp2*	1 (16.5)
	O164 (1)	*ompA, irp2, sitA*	1 (100)
	O167 (1)	*ompA, ompT, hra*	1 (100)
E {15 (29)}	O143 (10)	*ompA, traT, sitA, iha, chuA*	7 (70)
		*ompA, traT, iha, chuA*	2 (20)
		*ompA, traT, chuA*	1 (10)^b^
	O167 (5)	*ompA, traT, irp2, sitA, chuA*	4 (80)
		*ompA, traT, irp2, chuA*	1 (20)
B1 {9 (18)}	O28ac (3)	*ompA, traT, sitA, iha, fimA*	2 (66.6)
		*ompA, iha*	1 (33.3)
	O29 (5)	*ompA, traT, sitA,iha, fimA*	5 (100)
	O164 (1)	*ompA, irp2, sitA, iha, fimA*	1 (100)
B2 {1 (2)}	O164 (1)	*ompA,sitA, iha, chuA, fimA, kpMSTII, vat*	1 (100)
C {1 (2)}	O164 (1)	*ompA, kpsMTII*	1 (100)^c^

^a^VGs *afaBCIII, cvaC, iucD, iroN*, *papA, papC, sat, sfaDE,* and *α-hlyA* were not detected

^b^strain isolated in Chile

^c^strain received from CDC

### Presence of UPEC PAI genetic markers

All EIEC strains, regardless their phylogroup or serogroup, presented markers for at least one of eight pathogenicity islands searched (Table 3). PAI I_J96_ and PAI II_CFT073_ were detected at high frequencies (92.2% and 80.4%, respectively); whereas PAI IV_536_ and PAI I_CFT073_ were less frequent (37.2% and 31.4%, respectively). The majority of strains harbored multiple PAIs (two to four). In fact, strains harboring only one PAI were rarely found (one strain from group B2, two from group A, and three from group B1). It is worth mentioning that PAI I_CFT073_ was exclusively detected in all strains from phylogroups B2 and E. Besides, PAI IV_536_ was present only in strains from specific serogroups (O124, O152, O164, and O167) belonging to phylogroups A, B1, and E. All strains from EIEC serogroups O136, O143, O144, and O152 were uniforms in their content of PAIs. Interestingly, strains from serogroup O164 varied in their content of PAIs according to the phylogroup they were originated (Table 3).

**Table 3:** Distribution of UPEC pathogenicity islands in EIEC according to phylogroup and serogroup

Phylogroup {No. (%) of strains}	Serogroup (Total No. of strains)	No. (%) of strains	Pathogenicity Island^a^			
			I_J96_	II_CFT073_	IV_536_	I_CFT073_
A {25 (49)}	O124 (6)	4 (66.6)	+	+	+	-
		2 (33.3)	-	-	+	-
	O136 (6)	6 (100)	+	+	-	-
	O144 (5)	5 (100)	+	+	-	-
	O152 (6)	6 (100)	+	+	+	-
	O164 (1)	1 (100)	+	+	+	-
	O167 (1)	1 (100)	+	+	-	-
E {15 (29)}	O143 (10)	10 (100)	+	+	-	+
	O167 (5)	4 (80)	+	-	+	+
		1 (20)	+	+	+	+
B1 {9 (18)}	O28ac (3)	1 (33.3)	+	+	-	-
		1 (33.3)	+	-	-	-
		1 (33.3)	-	+	-	-
	O29 (5)	4 (80)	+	+	-	-
		1 (20)^b^	+	-	-	-
	O164 (1)	1 (100)	+	-	+	-
B2 {1 (2)}	O164 (1)	1 (100)	-	-	-	+
C {1 (2)}	O164 (1)^c^	1 (100)	+	+	-	-

^a^PAIs II_J96_, I_536_, II_536_, and III_536_ were not detected in any strain

^b^strain isolated in Chile

^c^strain isolated in Japan

## Discussion

EIEC is known to be restricted to a dozen serogroups [22, 23], all non-motile with very rare exceptions [21, 22, 24]. However, it has been demonstrated by molecular methods that non-motile EIEC strains present the genes for flagella [12, 25]. Although necessary for epidemiological assessment, molecular methods are not feasible in clinical laboratory settings in less developed geographical areas where negative tests for motility and lysine decarboxylation continue to be important phenotypic clues to direct the diagnosis of EIEC. It was interesting to note that biochemical behavior regarding lactose fermentation and indole production was phylogroup-related. The lack of indole production, although rarely reported among *E. coli* isolates [26], was frequently found among EIEC, remarkably associated to serogroups O124 and O136.

It has been accepted that diarrheagenic *E. coli* pathotypes are primordially originated from phylogenetic groups A, and B1 [27]. Nevertheless, prototype strains from important DEC pathovars are assigned to more virulent phylogroups as happens with EPEC strain E2348, and EHEC strain EDL933, which are originated from phylogroups B2 and E, respectively [19].

We have shown that EIEC strains analyzed in this study, do not have a unique phylogenetic origin. Instead, they have arisen most (67%) from phylogroups A, and E, while B1 origin was less common (18%). Moreover, the phylogenetic distribution showed to be strictly related to serogroup, since all O124, O136, O144, and O152 strains were assigned to phylogroup A, all O28ac, and O29 were B1, and all O143 and the majority of O167 were group E. Serogroup O164 was unique in presenting a flexible phylogenetic distribution, with strains spread in phylogroups A, B1, C, and B2. By using various molecular approaches, several authors have also shown the distribution of EIEC in different clusters [1, 28], or phylogroups [12]. In agreement with our results, Hazen et al. [12], using in silico analysis of complete genome sequences, showed that serogroup O124 was classified in phylogroup A, and O143 in phylogroup E. However, our results were discrepant from those authors in respect to serogroups O136, O144, and O164 that they assigned to group B1. One reason for this inconsistency might be the small number of strains sequenced in that study. Overall, and in accordance with other authors [1, 12, 28], the diversity in phylogenetic origin emphasizes the multiple evolutionary histories of EIEC. As mentioned, other DEC pathovars have already been demonstrated to exhibit diverse phylogenetic origin but, to our knowledge, the link between phylogeny and serogroup has not been covered in a comprehensive manner hitherto.

Although EIEC is a diarrheagenic pathotype, it showed to harbor two to seven out of 20 searched virulence genetic markers that are associated with virulence of ExPEC [29, 30]. The data presented in this study corroborate and further extend the idea that diarrheagenic pathotypes may share ExPEC VGs, and vice-versa [31–33]. In fact, some of the found VGs herein are widespread in *E. coli*, regardless the pathogenic potential of the strain.

Despite the low number of strains in each serogroup in this study, the presence of those VGs in EIEC may not be casual since the majority of strains (67 to 100%) presented specific virulence gene profiles for each of eight among nine serogroups studied. Further research is needed to understand the contribution of extraintestinal VGs to the pathogenesis of EIEC. Nonetheless, it is already known that the conjunction of virulence traits from diverse *E. coli* pathotypes may give rise to extreme virulent clones as have recently happened with *E. coli* O104:H4 which combined enteroaggregative and toxigenic properties [34].

Using the same methodology described by Sabaté et al. [35] this study detected four out of eight PAIs primarily described in UPEC. Interestingly, those authors reported 14% of UPEC missing all these PAIs, while all EIEC strains analyzed herein presented one to four PAIs. It was remarkable that PAI I_J96_ was the most frequent in EIEC (92%) while it was not detected in any of the commensal or UPEC isolates in that study. Other authors have also reported the absence or very low frequency of PAI I_J96_ in commensal and extraintestinal pathogenic *E. coli* isolates, including UPEC [36–39]. Island II_CFT073_ was detected in 80% of our EIEC isolates, and none were from phylogroup B2, while it was found by Sabaté [35] only in isolates from phylogroup B2. In agreement with Sabaté et al. [35], PAI I_CFT073_ was exclusively detected in EIEC strains from phylogroups B2 and E. Dobrindt et al. [40] reported the presence of PAIs I_536_ to IV_536_, or at least some of their DNA regions, in diarrheagenic isolates. Besides PAI IV_536_ no other PAIs from UPEC 536 were detected in this study. We have shown that EIEC also harbors multiple PAIs as described for UPEC [38]. However, the most predominant combination found in EIEC (PAIs I_J96_ and II_CFT073_) was not detected in UPEC [35, 38]. Except for PAI IV_536_ the other three PAIs detected in this study did not correlate with the presence of the recognized virulence traits they are reported to encode namely: α-hemolysin, P-fimbriae, Prs-fimbriae, cytotoxic necrotizing factor 1, and aerobactin [35]. The occurrence of DNA rearrangements in these PAIs could explain the missing virulence traits [40, 41], while other yet-unknown regions [42] could be perpetuated in EIEC. Recently reported a genomic analysis of EIEC strains [12, 17] had not mentioned the presence of sequences similar to UPEC PAIs although serogroups and phylogroups studied encompassed some of those analyzed herein. A possible explanation may be that those studies have focused only in virulence markers already known to be involved in the pathogenicity of EIEC and *Shigella* for the intestinal tract.

While extraintestinal virulence determinants tended to be segregated according to serogroup and phylogroup, the enteroinvasive determinants, either chromosomal or plasmid located, were maintained in all strains in this study, regardless their serogroup or phylogroup. This finding suggests that the enteroinvasive pathotype has evolved later in the EIEC evolution as pointed by others [12, 17].

Finally, as happens with the finding of ExPEC VGs, the presence of UPEC PAI genetic markers in EIEC needs to be further investigated to gain comprehension not only on the pathogenesis but also on the evolution of this important diarrheagenic pathotype.

## Conclusions

EIEC is a DEC pathovar that presents both VGs and PAI genetic markers typically found in ExPEC, especially UPEC. These features are distributed in a phylogenetic and serogroup-dependent manner suggesting the existence of stable EIEC subclones. The constant and uncommon presence of PAI I_J96_ suggests a not yet unveiled fact in the EIEC evolution which requires special attention. The data herein extends the observations reported in the current literature on the occurrence of heteropathogenic *E. coli*, meaning those strains that combine VGs specific of different *E. coli* pathovars.

The occurrence of clones combining virulence genes derived from different *E. coli* pathovars as a result of horizontal gene transfer could play a previously unsuspected role in the pathogenesis of EIEC infections.

## Methods

### Bacterial strains

A total of 51 enteroinvasive *E. coli* (EIEC) strains belonging to the bacterial collection of the Department of Microbiology, Immunology, and Parasitology (DMIP) at Federal University of São Paulo, were studied. The strains were isolated during a 24 years spanned period (between 1964 and 1987) from sporadic cases of human diarrhea in varied geographical regions, being 44 in São Paulo city (Southeast-Brazil), and four in Pernambuco state (North-Brazil). The other three isolates were received from Chile, Japan, and CDC (Centers for Disease Control and Prevention)-USA. Upon arrival at DMIP, strains were biotyped and serotyped by conventional methods [26] and submitted to the keratoconjunctivitis assay (Serény test) [43]. After characterization, the collection was kept in 15% glycerol at −70 °C, and in stabbed cultures at room temperature protected from light.

### Growth conditions and characterization

For this study, bacteria were grown in tryptic soy broth (TSB) (Difco Laboratories, Detroit, MI, USA), purified on MacConkey agar (Difco Laboratories, Detroit, MI, USA), and serogroup-confirmed by conventional methods [26] using specific antisera (Probac do Brasil- SP-Brasil). Biochemical behavior was assessed for the following reactions: gas from glucose, H_2_S production, urea hydrolysis, tryptophan deamination, lysine decarboxylation, motility, indole production, and Simmons citrate as sole carbon source, by using the identification kit enterokitB® (Probac do Brasil - SP-Brasil) as recommended.

### HeLa cells invasion assay

The invasion capacity was confirmed using cell culture assays as described by Keller et al. [44] with the following modifications: HeLa cells were used instead of Hep-2 cells, and incubation time after inoculation of bacteria was two hours, followed by another three hours in the presence of 50 μg/mL gentamicin. After saline wash, the coverslips containing the HeLa cells were fixed, stained and observed under light microscopy (x1,000 magnification).

### Plasmid profile

Plasmid profile was assessed using the rapid alkaline extraction method of Birnboim and Doly [45] exactly as described except that the volumes were scaled down as follows: 1 mL of broth culture, 100 μL of solution I, 200 μl of solution II, 150 μL of solution III, and 100 μL of solution IV. Electrophoresis was carried out during 130 min at 100–120 V and 40 mA in 0.8% agarose gel. Stained gels were visualized and recorded (Universal Hood II – Biorad Laboratories Inc., USA).

### Determination of phylogenetic origin

Determination of phylogenetic origin was carried out following the revised method described by Clermont et al. [19]. The method is based on a quadruplex PCR reaction to detect the genes *arpA*, *chuA, yjaA,* and TspE4.C2. The pattern of amplification for those four markers in that order defines the phylogroups as follows: A (+ − − -), B1 (+ − − +), B2 (− + + +) or (− + + −) or (− + − +), and F (− + − −). Strains presenting other amplification patterns were submitted to a second PCR reaction for the distinction between phylogroups A and C, D and E, or E and clade I according to Clermont et al. [19]. Prototype strains *E. coli* RS218, and EDL933 were used as positive controls. Reactions were performed in a Mastercycle Gradient Thermocycler (Mastercycle gradient – Eppendorf) using boiled isolated colonies as template DNA, primers from IDT (Integrated DNA Technologies - USA), and Go Taq Green Master Mix (Promega - USA). The resulted amplified DNA fragments were resolved on 2% agarose gels, stained and photographed in a digital image capture system (Universal Hood II – Biorad Laboratories, Inc. USA). “1Kb Plus DNA Ladder” (Invitrogen™ - Thermo Fisher Scientific) was used as a mass molecular marker.

### Search for ExPEC genetic virulence markers

EIEC strains were screened by PCR for 20 virulence genetic markers related to adhesins (*afaBCIII, fimA, hra, iha, papA, papC, sfaDE*), tissue invasin (*ompA*), iron capture systems (*chuA*, *iroN*, *irp2*, *iucD, sitA*), protectins (*cvaC, kpsMTII, ompT, traT*), and toxins (*α-hlyA, sat, vat*). The genes *iucD*, *irp2*, *vat*, and *fimA* were searched by single PCR reactions, and the other genes were searched by multiplex PCR reactions as presented in Table 4. The specific primer sequences, reaction conditions, amplicon sizes, and the control strains for each marker were described elsewhere [46].

**Table 4:** Detection of virulence genes by multiplex PCR^a^

	Gene (amplicon bp)	Annealing condition	Reference for multiplex composition
Multiplex 1	*afaBCIII* (750) *papC* (328) *sfaDE* (410)	65 °C – 1 min	[47]
Multiplex 2	*cvaC* (680) *KpsMT II* (272) *ompT* (559)	58 °C – 30 s	This study
Multiplex 3	*hlyA* (1,177) *iroN* (665) *papA* (720) *traT* (290)	58 °C – 30 s	This study
Multiplex 4	*ompA* (919) *hra* (537)	58 °C – 30 s	This study
Multiplex 5	*iha* (925) *sat* (667) *sitA* (608)	58 °C – 30 sec	This study

^a^primers for multiplex PCR reactions 2, 3, 4, and 5 were described elsewhere [46]. Genes *chu*A, *iuc*D*,irp2*, *vat*, and *fim*A were searched by single PCR reactions [46]

### Detection of pathogenicity islands

Pathogenicity islands described in UPEC strains CFT073 (PAIs I and II), J96 (PAIs I and II), and 536 (PAIs I, II, III, and IV) were searched by using the methodology defined by Sabaté et al. [35] except that PAIs II_CFT073_, III_536_, and IV_536_ were detected with single reactions. The PCR conditions were kept as described [35] and were the same for both single and multiplex reactions. As positive controls, we used UPEC prototype strain J96, NMEC prototype strain RS218 and bacteremic strain EC40 [46]. The primers used detect the following PAI-associated sequences or genes: RPA (PAI I_CFT073_); cft073.2Ent (PAI II_CFT073_); *pap*GI (PAI I_J96_); *hly*d and *cnf* (PAI II_J96_); I.9 and I.10 (PAI I_536_); orf1 (PAI II_536_); *sfa*A1 (PAI III_536_); *irp2* (PAI IV_536_) [35].

## Abbreviations

DEC: Diarrheagenic *E. coli*
ExPEC: Extraintestinal pathogenic *E. coli*
EIEC: Enteroinvasive *E. coli*
Kb: Kilobase
NMEC: Neonatal meningitis *E. coli*
PAI: Pathogenicity island
PAI I_J96_: Pathogenicity island I from UPEC strain J96
PAI II_J96_: Pathogenicity island II from UPEC strain J96
PAI I_CFT073_: Pathogenicity island I from UPEC strain CFT073
PAI II_CFT073_: Pathogenicity island II from UPEC strain CFT073
PAI I_536_: Pathogenicity island I from UPEC strain 536
PAI II_536_: Pathogenicity island II from UPEC strain 536
PAI III_536_: Pathogenicity island III from UPEC strain 536
PAI IV_536_: Pathogenicity island IV from UPEC strain 536
PCR: Polymerase chain reaction
pINV: Invasion plasmid
UPEC: Uropathogenic *E. coli*
VG(s): Virulence gene(s)

## References

[1] Lan R, Chehani Alles M, Donohoe K, Martinez MB, Reeves PR (2004). Molecular evolutionary relationships of enteroinvasive *Escherichia coli* and *Shigella* spp. Infect Immun..

[2] Toledo MRF, Trabulsi LR (1990). Frequency of enteroinvasive *Escherichia coli* in children with diarrhea and healthy controls in Sao Paulo, Brazil. Rev Microbiol Sao Paulo..

[3] Echeverria P, Sethabutr O, Serichantalergs O, Lexomboon U, Tamura K (1992). *Shigella* and enteroinvasive *Escherichia coli* infections in households of children with dysentery in Bangkok. J Infect Dis..

[4] Vieira N, Bates SJ, Soldberg OD, Ponce K, Howsmon R, Cevallos W (2007). High prevalence of enteroinvasive *Escherichia coli* isolated in a remote region of northern coastal Ecuador. Am J Trop Med Hyg..

[5] Tulloch EF, Ryan KJ, Formal SB, Franklin FA (1973). Invasive enteropathic *Escherichia coli* dysentery. An outbreak in 28 adults. Ann Intern Med.

[6] Snyder JD, Wells JG, Yashuk J, Puhr N, Blake PA (1984). Outbreak of invasive *Escherichia coli* gastroenteritis on a cruise ship. Am J Trop Med Hyg..

[7] Yamamura K, Sumi N, Egashira Y, Fukuoka I, Motomura S, Tsuchida R (1992). Food poisoning caused by enteroinvasive *Escherichia coli* (O164:H–) – a case in which the causative agent was identified. Kansenshogaku Zasshi..

[8] Nataro JP, Kaper JB (1998). Diarrheagenic *Escherichia coli*. Clin Microbiol Ver..

[9] Escher M, Scavia G, Morabito S, Tozzoli R, Maugliani A, Cantoni S (2014). A severe foodborne outbreak of diarrhoea linked to a canteen in Italy caused by enteroinvasive *Escherichia coli*, an uncommon agent. Epidemiol Infect..

[10] Silva RM, Toledo MR, Trabulsi LR (1982). Correlation of invasiveness with plasmid in enteroinvasive strains of *Escherichia coli*. J Infect Dis..

[11] Hale TL (1991). Genetic basis of virulence in *Shigella* species. Microbiol Rev..

[12] Hazen TH, Leonard SR, Lampel KA, Lacher DW, Maurelli AT, Rasko DA (2016). Investigating the relatedness of enteroinvasive *Escherichia coli* to other *E. coli* and *Shigella* isolates by using comparative genomics. Infect Immun.

[13] Harris JR, Wachsmuth IK, Davis BR, Cohen ML (1982). High-molecular-weight plasmid correlates with *Escherichia coli* enteroinvasiveness. Infect Immun..

[14] Kotloff KL, Winickoff JP, Ivanoff B, Clemens JD, Swerdlow DL, Sansonetti PJ (1999). Global burden of *Shigella* infections: implications for vaccine development and implementation of control strategies. Bull World Health Organ.

[15] Selander RK, Caugantu DA, Ochman H, Musser JM, Gilmour MH, Whittam TS (1986). Methods of multilocus enzyme electrophoresis for bacterial population genetics and systematics. Appl Environ Microbiol.

[16] Maiden MCJ, Bygraves JA, Feil E, Morelli G, Russel J, Urwin R (1998). Multilocus sequence typing: a portable approach to the identification of clones within populations of pathogenic microorganisms. Proc Natl Acad Sci USA.

[17] Pettengill EA, Pettengill JB, Binet R (2016). Phylogenetic analyses of *Shigella* and enteroinvasive *Escherichia coli* for the identification of molecular epidemiological markers: whole-genome comparative analysis does not support distinct genera designation. Front Microbiol..

[18] Clermont O, Bonacorsi S, Bingen E (2000). Rapid and simple determination of the *Escherichia coli* phylogenetic group. Appl Environ Microbiol..

[19] Clermont O, Christenson JK, Denamur E, Gordon DM (2013). The Clermont *Escherichia coli* phylo-typing method revisited: improvement of specificity and detection of new phylo-groups. Envir Microb Rep..

[20] Ochman H, Selander RK (1984). Evidence for clonal population structure in *Escherichia coli*. Proc Nat Acad Sci USA.

[21] Michelacci V, Prosseda G, Maugliani A, Tozzoli R, Sanchez S, Herrera-León S (2016). Characterization of an emergent clone of enteroinvasive *Escherichia coli* circulating in Europe. Clin Microbiol Infect.

[22] Silva RM, Toledo MR, Trabulsi LR (1980). Biochemical and cultural characteristics of invasive *Escherichia coli*. J Clin Microbiol.

[23] Kaper JB, Nataro JP, Mobley HLT (2004). Pathogenic *Escherichia coli*. Nat Rev Microbiol..

[24] Gomes TA, Toledo MR, Trabulsi LR, Wood PK, Morris JG (1987). DNA probes for identification of enteroinvasive *Escherichia coli*. J Clin Microbiol..

[25] Amhaz JM, Andrade A, Bando SY, Tanaka TL, Moreira-Filho CA, Martinez MB (2004). Molecular typing and phylogenetic analysis of enteroinvasive *Escherichia coli* using the *fli*C gene sequence. FEMS Microbiol Lett..

[26] Ewing WH (1986). Edwards and Ewing’s identification of *Enterobacteriaceae*.

[27] Boyd EF, Hartl DL (1998). Chromosomal regions specific to pathogenic isolates of *Escherichia coli* have a phylogenetically clustered distribution. J Bacteriol..

[28] Bando SY, do-Valle GR, Martinez MB, Trabulsi LR, Moreira-Filho CA (1998). Characterization of enteroinvasive *Escherichia coli* and *Shigella* strains by RAPD analysis. FEMS Microbiol Lett..

[29] Johnson JR, Russo TA (2005). Molecular epidemiology of extraintestinal pathogenic (uropathogenic) *Escherichia coli*. Inter J Med Microbiol..

[30] Ewers C, Li G, Wilking H, Kiebling S, Alt K, Antáo EM (2007). Avian pathogenic, uropathogenic, and newborn meningitis-causing *Escherichia coli*: how closely related are they?. Inter J Med Microbiol..

[31] Olesen B, Scheutz F, Andersen RL, Menard M, Boisen N, Johnston B (2012). Enteroaggregative *Escherichia coli* O78:H10, the cause of an outbreak of urinary tract infection. J Clin Microbiol..

[32] Abe CM, Salvador FA, Falsetti IN, Vieira MA, Blanco J, Blanco JE (2008). Uropathogenic *Escherichia coli* (UPEC) strains may carry virulence properties of diarrheagenic *E. coli*. FEMS Immunol Med Microbiol.

[33] Kessler R, Nisa S, Hazen TH, Horneman A, Amoroso A, Rasko DA (2015). Diarrhea, bacteremia and multiorgan dysfunction due to an extraintestinal pathogenic *Escherichia coli* strain with enteropathogenic *E. coli* genes. Pathog Dis.

[34] Beutin L, Martin A (2012). Outbreak of Shiga toxin-producing *Escherichia coli* (STEC) O104:H4 infection in Germany causes a paradigm shift with regard tohuman pathogenicity of STEC strains. J Food Prot..

[35] Sabaté M, Moreno E, Perez T, Andreu A, Prats G (2006). Pathogenicity island markers in commensal and uropathogenic *Escherichia coli* isolates. Clin Microbiol Infect..

[36] Li B, Sun JY, Han LZ, Huang XH, Fu Q, Ni YX (2010). Phylogenetic groups and pathogenicity island markers in fecal *Escherichia coli* isolates from asymptomatic humans in China. Appl Environ Microbiol..

[37] Koga VL, Tomazetto G, Cyoia PS, Neves M, Vidotto MC, Nakazato G. Molecular screening of virulence genes in extraintestinal pathogenic *Escherichia coli* isolated from human blood culture in Brazil. Biomed Res Int. 2014;9:1–9. 10.1155/2014/465054 PMC4009324 24822211

[38] Cyoia PS, Rodrigues GR, Nishio EK, Medeiros LP, Koga VL, Pereira AP (2015). Presence of virulence genes and pathogenicity islands in extraintestinal pathogenic *Escherichia coli* isolates from Brazil. J Infect Dev Ctries..

[39] Navidinia M, Najar-Peerayeh S, Fallah F, Bakhshi B, Adabian S (2013). Distribution of pathogenicity islands markers (PAIs) in uropathogenic *Escherichia coli* isolated from children in Mofid Children Hospital. Arch Pediatr Infect Dis..

[40] Dobrindt U, Blum-Oehler G, Nagy G, Schneider G, Johann A, Gottschalk G (2002). Genetic structure and distribution of four pathogenicity islands (PAI I536 to PAI IV536) of uropathogenic *Escherichia coli* strain 536. Infect Immun..

[41] Dobrindt U, Hacker J (2001). Whole genome plasticity in pathogenic bacteria. Curr Opin Microbiol..

[42] Schmidt H, Hensel M (2004). Pathogenicity islands in bacterial pathogenesis. Clin Microbiol Rev..

[43] Serény B (1955). Experimental *Shigella* keratoconjunctivitis. A preliminary report. Acta Microbiol Acad Sci Hung.

[44] Keller R, Pedroso MZ, Ritchmann R, Silva RM (1998). Occurrence of virulence-associated properties in *Enterobacter cloacae*. Infect Immun..

[45] Birnboim HC, Doly J (1979). A rapid alkaline extraction procedure for screening recombinant plasmid DNA. Nucleic Acids Res..

[46] Santos ACM, Zidko ACM, Pignatari AC, Silva RM (2013). Assessing the diversity of virulence potential of *Escherichia coli* isolated from bacteremia in São Paulo, Brazil. Braz J Med Biol Res..

[47] Le Bouguenec C, Archambaud M, Labigné A (1992). Rapid and Specific Detection of the *pap*, *afa*, and *sfa* adhesin-encoding operons in uropathogenic *Escherichia coli* strains by polymerase chain reaction. J Clin Microbiol..

